# Therapeutic potential of curcumin in regenerative dentistry

**DOI:** 10.3389/fdmed.2025.1537478

**Published:** 2025-03-24

**Authors:** Anahid A. Birjandi, Paul Sharpe

**Affiliations:** Centre for Craniofacial and Regenerative Biology, Faculty of Dentistry, Oral & Craniofacial Sciences, Kings College London, London, United Kingdom

**Keywords:** dental pulp, curcumin, tetrahydrocurcumin, tissue regeneration, natural therapeutic

## Abstract

**Introduction:**

Natural compounds have emerged as promising candidates in drug development due to their potent immunomodulatory anti-inflammatory, antibacterial, analgesic, and healing properties. They have shown significant therapeutic potential in clinical applications, such as mouth rinses, toothpastes, and localized delivery systems. The use of natural alternatives can contribute to tackling antimicrobial resistance. Among natural compounds, curcumin has gained particular attention, demonstrating robust anti-cancer, antibiotic, and anti-inflammatory activities in numerous *in vivo* studies, while exhibiting a favorable safety profile for the treatment of various diseases. In this study, the remedial effects of curcumin and its metabolite, tetrahydrocurcumin, on dental pulp were explored. In addition, these results were compared with our previous findings on the effects of these natural compounds on periodontal ligament and gingival epithelial cells, further broadening our understanding of their therapeutic potential in oral disease such as caries and periodontitis.

**Methods:**

RNA sequencing was used to investigate the differentially expressed genes in dental pulp cells following treatments with curcumin and tetrahydrocurcumin.

**Results:**

We show that treatment of dental pulp cells with 1 μM of curcumin or tetrahydrocurcumin is sufficient to promote Wnt signaling pathway in dental pulp cells. Curcumin treatment promotes the upregulation of cellular metabolism and enhances cellular response to stress. Our enrichment analysis shows that treatment with tetrahydrocurcumin modulates the extracellular matrix and angiogenesis.

**Conclusions:**

The findings of this study highlight the cytoprotective and regenerative properties of curcumin and tetrahydrocurcumin. These properties could be leveraged as a therapeutic approach to promote tissue regeneration in oral diseases.

## Introduction

Natural compounds play a vital role in modern medical practices and health strategies due to their potential in modulating inflammation, oxidative stress, and cellular signaling pathways (1). Modern extraction techniques have improved the stability and bioavailability of many herbal compounds, making them even more effective for clinical applications (2). They can be used in treating chronic diseases such as diabetes, neurodegenerative disorders, cardiovascular conditions, and cancer (3–5). Incorporation of herbal medicine into clinical practices allows for the investigation of synergistic interactions between traditional remedies and modern pharmaceuticals (6–8).

Recent advances in regenerative dentistry are revolutionizing oral healthcare. Mesenchymal stem cells (MSCs) derived from dental pulp, periodontal ligament, or bone marrow possess remarkable potential for differentiating into various cell types, such as osteoblasts, odontoblasts, and fibroblasts (9–11). These properties enable their application in regenerating damaged oral tissues. In addition, the use of hydrogels and nanomaterials to support cell growth and differentiation enhances the precision and success of tissue regeneration. However, regulatory considerations, cost, and long-term stability remain challenging factors in incorporating these advances in the clinical setting (12–17). Natural availability, affordability, and the relatively low toxicity of some natural compounds render them a potential alternative in regenerative approaches. Multiple independent studies have demonstrated wound healing and anti-inflammatory properties of these compounds *in vivo* (18–21). As an example, citrus extract exhibits antibacterial, anti-inflammatory, and tissue-regenerative characteristics (22). Bioactive compounds, such as polyphenols, terpenoids, alkaloids, saponins, and peptides, have been shown to be efficient in the management of periodontal disease (23). Curcumin, a polyphenolic compound derived from the rhizome of *Curcuma longa*, has gained scientific interest for its diverse pharmacological properties, including anti-inflammatory, antioxidant, and regenerative effects. Curcumin exerts its regenerative effects primarily by modulating signaling pathways crucial for cell proliferation, differentiation, and survival. It can regulate signaling pathways, such as Wnt/β-catenin and PI3K/Akt/mTOR, and enhance stem cell viability and differentiation in neural repair (4, 5). In skin regeneration, curcumin has been shown to promote fibroblast migration and angiogenesis, which are key processes for wound healing (4). In bone regeneration, curcumin enhances osteoblast differentiation and mineralization by modulating RANKL/OPG signaling and reducing oxidative stress-induced damage (4, 6). Despite anti-inflammatory and pro-regenerative properties of curcumin, poor bioavailability and rapid metabolism and elimination of this compound have led to its limited clinical application. Tetrahydrocurcumin (THC) is one of the major metabolites of curcumin. The chemical structure of curcumin and THC are very similar, with THC lacking the double bonds that exist in the central seven-carbon chain of curcumin. THC is reported to have superior bioavailability than curcumin and exhibit a different molecular mechanism and biological activity. THC exhibits cytoprotective and anti-oxidative properties and has been proposed to be more potent than curcumin in the treatment of various conditions (24–26). In this study, the impact of curcumin and THC treatment on dental pulp was investigated. Using RNA sequencing, differentially expressed genes in human dental pulp cells were investigated to find an effective natural alternative in regenerative approaches in oral cavities.

## Methods

### Cell culture

Primary human dental pulp cells used in these assays were a kind donation from Dr. Ana Angelova at King's College London. Original cell harvest experiments were undertaken with the understanding and written consent of each individual and in full accordance with the World Medical Association Declaration of Helsinki (version 2002). The study was approved and followed the guidelines set by the Ethical Committee for human studies at King's College Hospital, King's College University of London. These cells were expanded in Full DMEM media with 10% fetal bovine serum (FBS).

### Viability

Curcumin and tetrahydrocurcumin compounds were provided by Colgate Palmolive. For viability, 20,000 cells/cm^2^ dental pulp cells were plated in triplicates in 96-well plates and incubated at 37°C, 5% CO_2_ for 24 h. Curcumin and tetrahydrocurcmin were used at concentrations 100 μM, 50 μM, 10 μM, 1 μM and 0.5 μM to treat dental pulp cells. As the stock of these compounds was prepared in dimethylsulfoxide (DMSO), DMSO was used as the vehicle-only control in all the experiments in addition to the media-only control. Cell viability was assessed using an MTS Assay Kit (Cell Proliferation, Colorimetric, ab197010). A colorimetric plate reader (Thermo Multiskan Ascent 354 microplate reader) was used to read the absorbance at 490 nm. Normal distribution of results was tested using the Shapiro–Wilk test. Statistical significance against 100% viability in media was reported using a one-way ANOVA and Dunnett's multiple comparisons in GraphPad Prism 8.3.0. The adjusted *p*-value is reported in graphs according to *New England Journal of Medicine* guidelines: *p* < 0.001 (***), *p* < 0.002 (**), *p* < 0.033 (*), and *p* > 0.12 (ns).

### Quantitative polymerase chain reaction (qPCR)

Dental pulp cells were plated at 0.05 × 10^6^ cells in triplicates in 24-well plates and incubated for 24 h (37°C, 5% CO_2_/95% air, 100% humidity) using a standard culture medium. Media only and DMSO (vehicle only) were used as the negative and vehicle controls, respectively. 6-Bromoindirubin-3′-oxime (BIO), a known Wnt pathway activator inhibitor, was used as a positive control at 50 nM (Sigma, St. Louis, MO, USA). After 24 h of treatment with 1 µM of curcumin, tetrahydrocurcumin, and controls, cells were lysed with Trizol for RNA extraction. RNA was reverse transcribed using random primers (M-MLV Reverse Transcriptase kit; Promega, Madison, WI, USA) according to the manufacturer's instructions. Gene expression was then assayed by real-time qPCR using SYBR Green (Roche, Basel, Switzerland) on a Rotor-Gene Q cycler (Qiagen, Hilden, Germany) system. Beta-actin was used as a housekeeping gene (Forward-GGCTGTATTCCCCTCCATCG, Reverse-CCAGTTGGTAACAATGCCTGT) and Axin2 as the read-out for Wnt pathway activity (Forward-TGACTCTCCTTCCAGATCCCA, Reverse-TGCCCACACTAGGCTGACA). Reactions were performed in triplicate and relative changes to housekeeping gene expression were calculated using the 2−ΔΔCT method, where CT is the threshold cycle. Groups were then analyzed with one-way ANOVA followed up with multiple comparison tests in GraphPad Prism 8. The adjusted *p*-value is reported in graphs according to the *New England Journal of Medicine* guidelines: *p* < 0.001 (***), *p* < 0.002 (**), *p* < 0.033 (*), and *p* > 0.12 (ns).

### Bulk sequencing

Dental pulp cells were seeded at a density of 0.05 × 10^6^ in triplicate and treated with freshly made 1 µM curcumin and tetrahydrocurcumin for 24 h. RNA was isolated using Trizol and Qiagen Mini Kit (Qiagen, Germany). After assessment of quality and quantity, RNA was sent for bulk sequencing. Partek RNA sequencing pipeline was used. All algorithms used to analyze the data were rerun with default settings, unless otherwise indicated. The quality of the sequencing reads was examined using FastQC (v0.11.4) (https://www.bioinformatics.babraham.ac.uk/projects/fastqc/). Raw sequencing reads (100-nt, paired-end) were trimmed using Trim Galore! (v1.001.001) (https://www.bioinformatics.babraham.ac.uk/projects/trim_galore/). Traces of ribosomal RNA and mitochondrial RNA were removed using Bowtie2 (v2.2.5) (27). Reads were aligned to the human reference genome GRCh38 using STAR (v2.7.3a) aligner with multi-sample setting (28). Mapping and alignment quality were examined using FastQC and duplicate reads were removed using the Mark Duplicates function of the Picard tools (v2.17.11) (http://broadinstitute.github.io/picard/). Reads were annotated using the Partek E/M with Ensembl Transcripts release 104. Samples were then visualized and explored using unsupervised methods and clustered based on principal component analysis (PCA), UMAP, tSNE, and hierarchical clustering. Differentially expressed genes (DEGs) were analyzed between media controls versus curcumin or tetrahydrocurcumin using DESeq 2 (v3.5) (29). DEGs with FDR value ≤0.05 and fold change ≥1.5 were filtered out for further analysis. For gene ontologies, Enrichr biological function and bioplanet pathways were used. For functional enrichment analysis, G profiler was used with a significance threshold set at Bonferroni correction and the term size set at 50. For the analysis of shared upregulated genes in pulp, periodontal ligament (PDL), and gingival epithelial cells, we looked at the DEG from our previous study (30) and compared it to the DEG from the dental pulp cells.

## Results

### Curcumin and tetrahydrocurcumin promote Wnt signaling pathway in dental pulp cells

**Figure 1:**
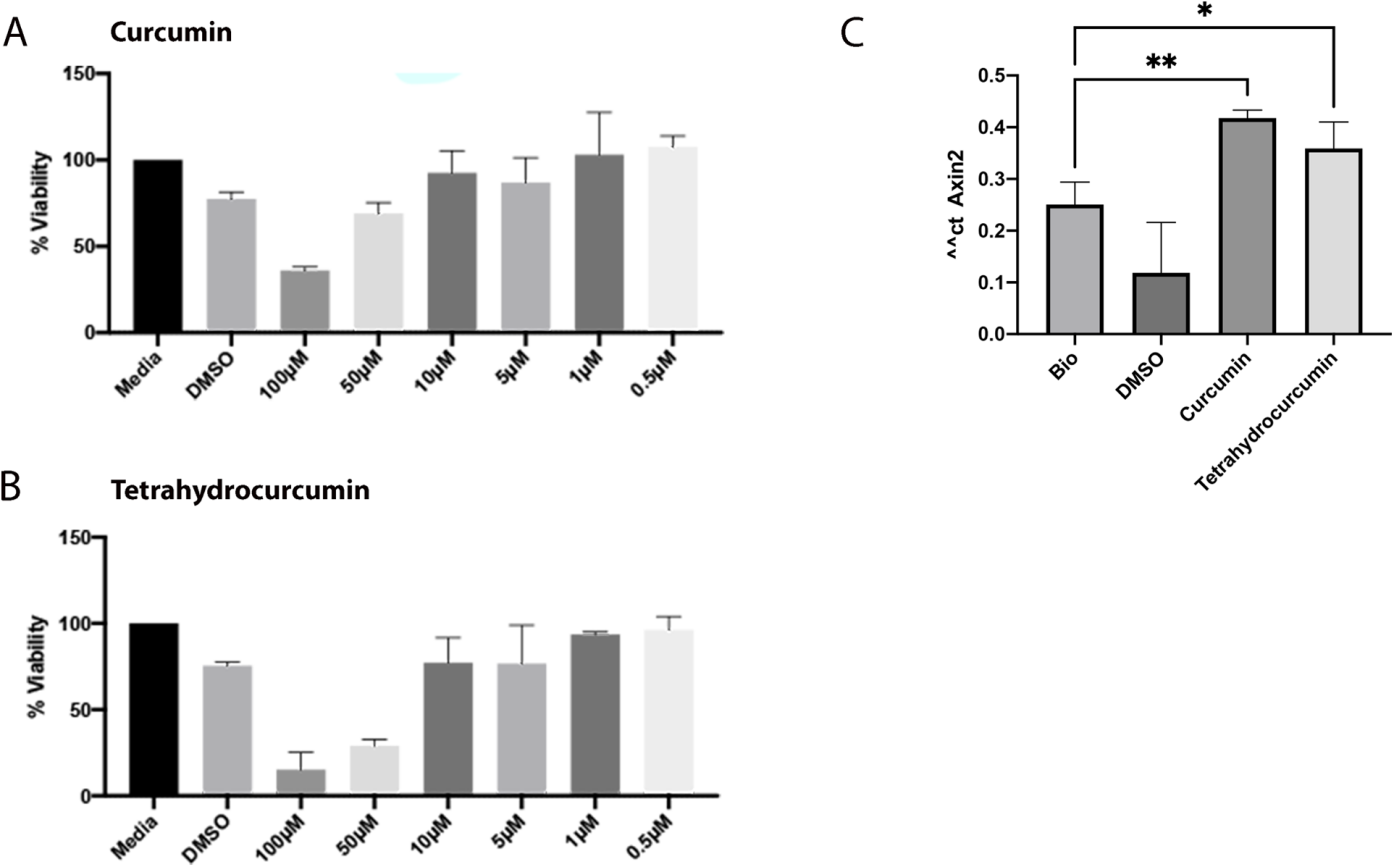
Effect of curcumin and tetrahydrocurcumin on viability of dental pulp cells and promotion of Wnt signaling pathway. MTS assay of dental pulp with concentrations of 100 μM, 50 μM, 10 μM, 1 μM and 0.5 μM of curcumin (**A**) and tetrahydrocurcumin (**B**). Pulp cells were treated with DMSO, 50 nM Bio, and 1 µM concentration of curcumin and tetrahydrocurcumin. Increased *Axin2* expression was detected after treatment with curcumin and tetrahydrocurcumin (*p*-value < 0.0001) (**C**).

To evaluate the impact of curcumin and tetrahydrocurcumin on pulp, the effect of these compounds on dental pulp cell viability was assessed at concentrations of 100 μM, 50 μM, 10 μM, 1 μM and 0.5 µM (Figure 1). At 1 and 0.5 µM, both compounds resulted in more than 90% viability of pulp cells. Therefore, 1 µM was selected as the optimum concentration for further downstream analysis. Wnt signaling pathway is crucial in the development and regeneration of many tissues in the oral cavity. *Axin2* is a downstream target of this pathway, and its expression is commonly used as a read-out of the level of Wnt signaling pathway activation (31, 32). Therefore, *Axin2* expression level analysis showed that 1 μM of curcumin and THC can promote Wnt signaling pathway in dental pulp cells.

### Curcumin and tetrahydrocurcumin promote matrix organization and multiple signaling pathways in dental pulp cells

**Figure 2:**
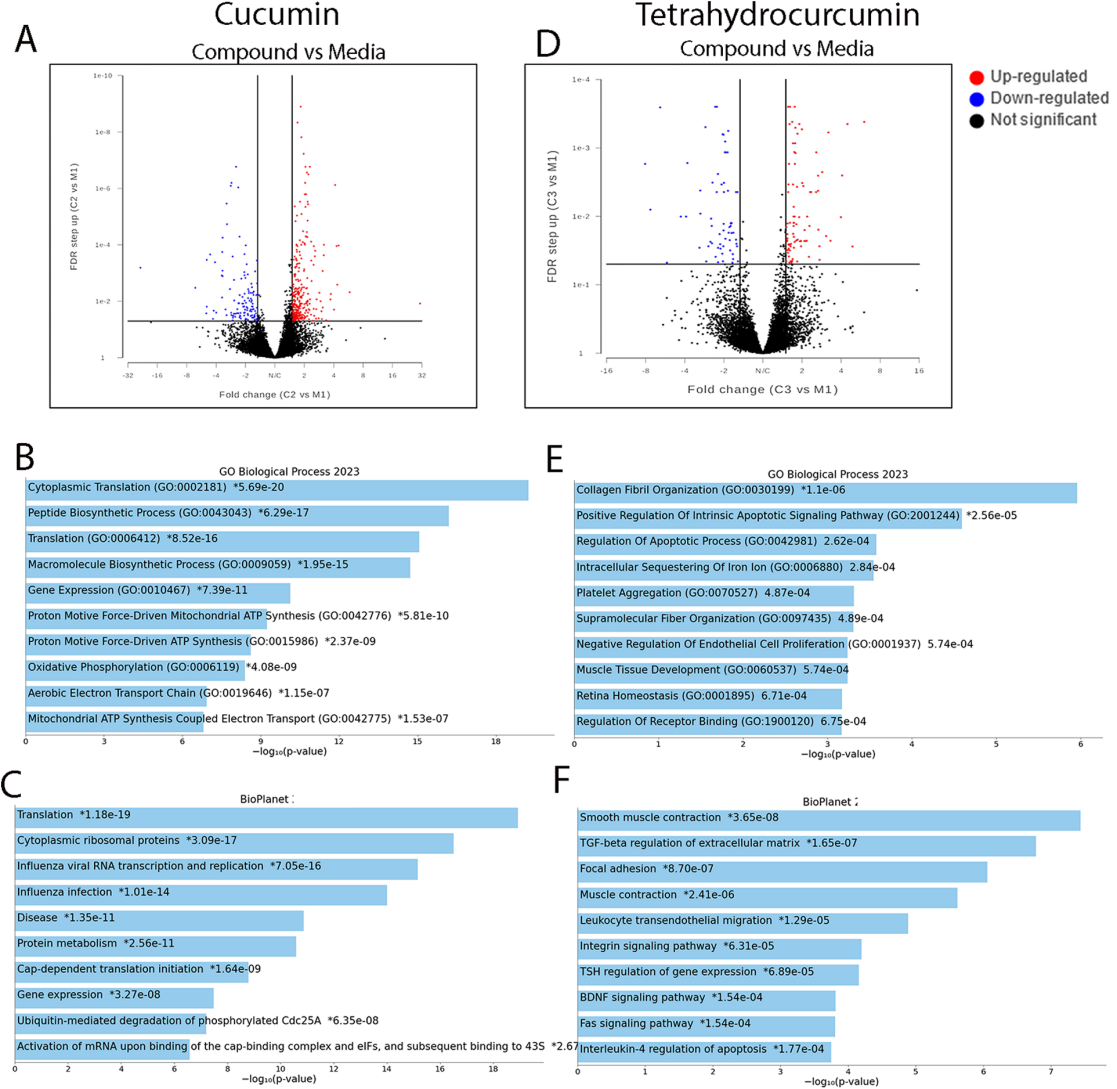
Differential gene expressions after treatment of pulp cells with curcumin and tetrahydrocurcumin. Volcano plots of differentially expressed genes after treatment of pulp cells with curcumin (**A**). Gene ontology (GO) of selected biological function (**B**) and pathways (**C**) in curcumin treatment at FDR value ≤0.05 and fold change ≥1.5. Volcano plots of differentially expressed genes after treatment of pulp cells with curcumin (**D**). GO of selected biological function (**E**) and pathway (**F**) in treatment with tetrahydrocurcumin (**F**) at FDR value ≤0.05 and fold change ≥1.5.

After determining the suitable concentration of curcumin and tetrahydrocurcumin for the treatment of pulp cells, 1 µM of these compounds was used to investigate the DEGs. Media and DMSO treatments were used as controls (see Supplementary Material). At an FDR value ≤0.05 and fold change ≥1.5, treatment with curcumin as well as tetrahydrocurcumin resulted in more than 100 DEGs (Figure 2). Curcumin treatment resulted in the upregulation of cytoplasmic translation, gene expression, macromolecule biosynthesis, and oxidative phosphorylation. Treatment with tetrahydrocurcumin resulted in the upregulation of collagen fibril organization, regulation of apoptosis, platelet aggregation, and supramolecular fiber organization and upregulation of many signaling pathways, such as TGFB, Integrin, FAS, BDNF, and IL4 regulation of apoptosis.

### Shared upregulated genes in pulp, PDL, and gingival cells after treatment with curcumin and tetrahydrocurcumin

**Figure 3:**
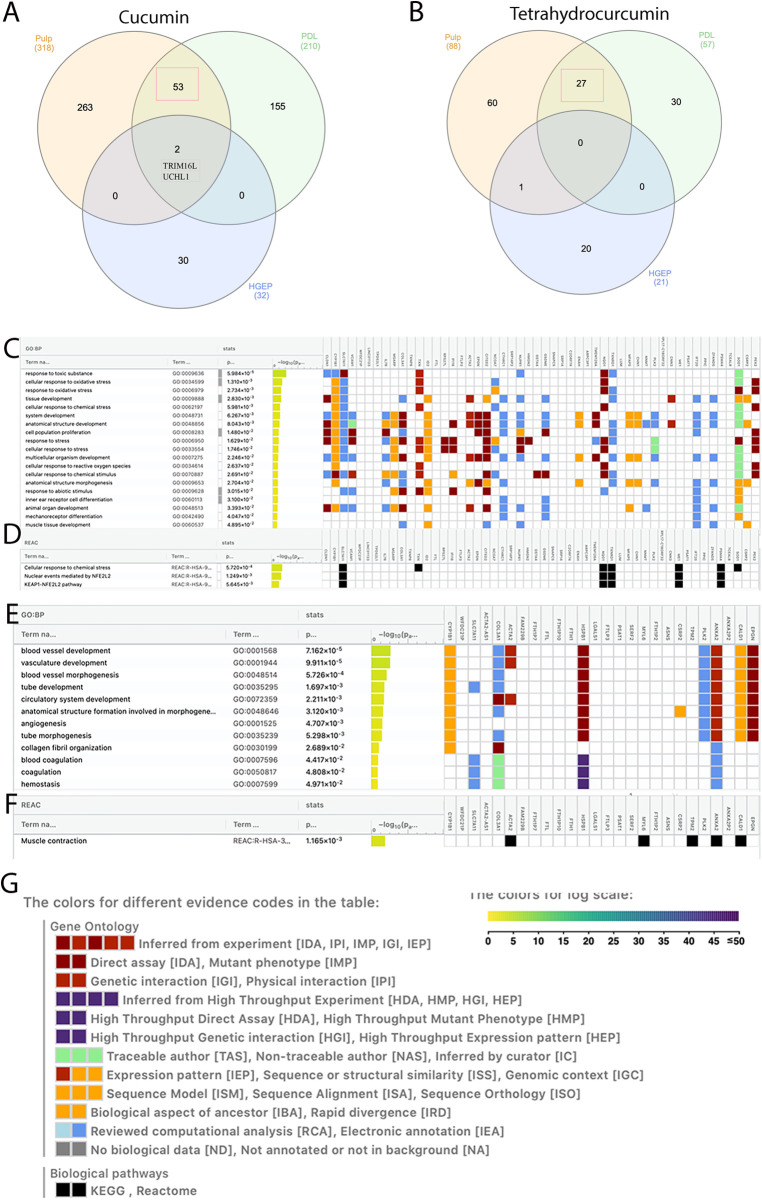
Shared and unique upregulated genes in pulp, periodontal ligament (PDL), and gingival epithelial cells. Venn diagram showing shared and unique upregulated genes after treatment of pulp, PDL, and human ginigval epithelial cells (HGEP) cells with curcumin (**A**) and tetrahydrocurcumin (**B**). Gene ontology (GO) enrichment analysis by g: profiler of the shared upregulated genes in pulp and PDL after treatment with curcumin demonstrates biological processes (**C**) and reactome (**D**). GO enrichment analysis by g: profiler of the shared upregulated genes in pulp and PDL cells after treatment with tetrahydrocurcumin demonstrates biological processes (**E**) and reactome (**F**). Graphical illustration guide (**G**).

We previously showed the impact of curcumin and tetrahydrocurcumin on PDL and gingival cells (30). Therefore, we asked if there are any shared upregulated genes between human dental pulp cells, PDL, and human gingival epithelial cells (HGEP) upon treatment with curcumin and tetrahydrocurcumin. To do this, all upregulated genes in these three cell types at an FDR value ≤0.05 and fold change ≥1.5 (Figure 3) were analyzed. Treatment with curcumin results in the highest number of upregulated genes in pulp cells. There were 53 shared upregulated genes between pulp and PDL. Gene ontology (GO) enrichment analysis of these shared genes using g: profiler demonstrates enrichment in response to toxic substance and oxidative stress, tissue development, and cell proliferation. UCHL1 and Tripartite Motif Containing 16 Like (Pseudogene) (TRIM16l) were upregulated in all cell types upon treatment with curcumin. Treatment with tetrahydrocurcumin results in the highest number of upregulated genes in pulp cells and 27 shared upregulated genes between pulp and PDL. GO enrichment analysis of these shared genes using g: profiler demonstrates enrichment in angiogenesis, coagulation, and muscle contraction. There were no shared upregulated genes between pulp, PDL, and gingival epithelial cells upon treatment with tetrahydrocurcumin.

## Discussion

We previously demonstrated anti-inflammatory and regenerative potential of curcumin and tetrahydrocurcumin on human PDL and gingival epithelial cells (30). In this study, we investigated the regenerative potential of curcumin and tetrahydrocurcumin on dental pulp cells. Our results show that 1 µM of curcumin and tetrahydrocurcumin promote Wnt signaling pathway in dental pulp cells without adverse effects on viability, similar to their effect on PDL and gingival epithelial cells. Wnt signaling pathway is associated with response to tissue damage (33–38), suggesting a regenerative potential of curcumin and its metabolite on dental pulp cells.

RNA sequencing demonstrates that treatment of dental pulp cells with curcumin results in upregulation of peptide biosynthetic process, macromolecule biosynthesis, mitochondrial ATP synthesis, cytoplasmic translation, and oxidative phosphorylation, suggesting a role in cellular metabolism and cytoprotection. Effect of curcumin on mitochondrial biogenesis has been shown previously. Recent studies have highlighted a multifaceted role for curcumin in cellular metabolism. This is through modulation of several metabolic pathways: scavenging reactive oxygen species (ROS), increasing antioxidant enzyme activity, regulating energy metabolism, and influencing the AMP-activated protein kinase (AMPK) pathway (39, 40). When compared to its impact on PDL and gingival epithelial cells, treatment with curcumin resulted in a higher number of upregulated genes in pulp cells. Shared upregulated genes between pulp and PDL were enriched in response to toxic substance and oxidative stress, tissue development, and cell proliferation. This suggests that curcumin has a role in cytoprotection and modulation of the cellular response to stress, such as inflammation and oxidative damage in oral tissues. Interestingly, TRIM16l and UCHL1 were upregulated in dental pulp, PDL, and gingival epithelial types upon treatment with curcumin. TRIM16 plays significant roles in cellular processes such as protein homeostasis, autophagy, and regulation of cellular responses to stress. This is particularly important for its involvement in the management of protein aggregates under oxidative or proteotoxic stress, especially in conditions like neurodegeneration and cancer (41, 42). UCHL1, a mitochondrial 10-formyltetrahydrofolate dehydrogenase, is an important mediator of many cellular functions and bone and lipid metabolism (43–47). It has recently been shown that UCLH1 can mediate MAPK signaling pathway in orthodontic tooth movement (48). Ubiquitination is a post-translational modification that affects many cellular pathways, such as immune response, angiogenesis, cell proliferation, apoptosis, and DNA repair. Deubiquitinating enzymes reversely modify proteins (49). Ubiquitination and deubiquitination play key roles in many diseases such as cancer (50). Interestingly, UCLH1 plays an important role in deubiquitination and TRIM16 can promote ubiquitination of proteins (47, 51). Upregulation of UCHL1 in pulp, PDL, and gingival epithelial cells upon curcumin treatment demonstrates that this natural compound has an important role in maintaining cellular proteostasis, particularly under stress conditions, making it a potential natural preventative and therapeutic candidate for oral diseases.

Treatment of dental pulp cells with tetrahydrocurcumin resulted in collagen fibril organization, platelet aggregation, regulation of apoptotic signaling pathway, and intracellular sequestering of iron ion. Signaling pathways such as TGFB, Integrin, FAS, BDNF, and IL4 regulation of apoptosis are also upregulated upon treatment with tetrahydrocurcumin. Integrins are expressed in dental pulp and play a role in cell attachment on extracellular matrix proteins (52, 53). Integrin a5 promotes odontogenetic differentiation of dental pulp stem cells due to extracellular matrix deposition (54). Integrins also have a role in dental pulp stem cell senescence through mTOR signaling pathway (55). FAS pathway has been shown to modulate apoptosis and regulate immunomodulation in dental pulp stem cells (56, 57). BDNF has a role in inflammation and can mediate odontoblasts differentiation (58). Upregulation of these pathways in THC treatment suggests a role in tissue remodeling and regeneration for tetrahydrocurcumin in dental pulp cells. Furthermore, shared upregulated genes between pulp and PDL after tetrahydrocurcumin treatment were enriched in angiogenesis and coagulation, suggesting a regenerative potential of this natural compound on dental pulp cells.

Regenerative potential of curcumin has been shown in multiple independent studies (59–64). Curcumin plays an anti-inflammatory role in dental pulp stem cells and can regulate proliferation and senescence of dental follicle cells. Curcumin-loaded biomaterials have been developed to enhance tissue regeneration and reduce local inflammation (65–67). However, curcumin's poor bioavailability has led to innovative approaches to improve absorption and enhance its effectiveness *in vivo* (68). Tetrahydrocurcumin, with its improved bioactivity, has shown promise in preclinical models of ischemic injury, enhancing angiogenesis while reducing fibrosis and oxidative damage (4, 6). Similarly, anti-inflammatory properties of tetrahydrocurcumin have been shown in independent studies. This natural compound can reduce the release of TNF-α and IL-6 and influence other inflammatory pathways, such as NF-*κ*B, JAK/STAT, and MAPK, which are activated in chronic diseases and metabolic disorders (4, 5). Tetrahydrocurcumin has been shown to be neuroprotective and pro angiogenesis and exert vascular protection in the brain (69–72). Interestingly, THC has been shown to suppress angiogenesis in murine adipose tissue and in human osteosarcoma (73, 74). This suggests that the impact of tetrahydrocurcumin on angiogenesis may be tissue dependent and can be harnessed to promote tissue regeneration or suppress tissue invasion and metastasis. Notably, THC has also been shown to be effective in the treatment of cancer (75). The results of this study show that curcumin and tetrahydrocurcumin promote Wnt signaling pathway in dental pulp, suggesting a role in tissue development and regeneration. In our study, curcumin exhibits cytoprotective properties and can regulate cellular metabolism and response to stress in dental pulp. The wider benefits of curcumin in cellular response to stress in oral tissue can be proposed by the upregulation of UCHL1 in dental pulp, PDL, and gingival epithelial cells, suggesting that curcumin can serve as a natural prophylactic and therapeutic candidate for pulp and PDL. Tetrahydrocurcumin affects different signaling pathways in dental pulp. It can promote regulation of the extracellular matrix, angiogenesis, and apoptotic signaling, suggesting a role in homeostasis and tissue regeneration. Enrichment in blood vessel development and morphogenesis in pulp and PDL after tetrahydrocurcumin treatment suggests this natural compound can serve as a candidate for oral wound healing.

## Conclusion

The results of this study suggest that curcumin and tetrahydrocurcumin exhibit cytoprotective and regenerative potential on oral tissue. Investigation of differentially expressed genes provides a comprehensive analysis of affected biological processes and detection of any adverse effect these compounds may have. To overcome the limitations of bulk RNA sequencing and fully recognize the therapeutic potential of these compounds in treating oral diseases, further studies and *in vivo* validation are recommended.

## Data Availability

RNASeq datasets generated and/or analyzed during the current study are available in the NCBI repository with accession number PRJNA1192542.

## References

[1] Li W Xiang Z Yu W Huang X Jiang Q Abumansour A Natural compounds and mesenchymal stem cells: implications for inflammatory-impaired tissue regeneration. Stem Cell Res Ther. (2024) 15(1):34. 10.1186/s13287-024-03641-3 38321524 PMC10848428

[2] Yadav S Mali SN Pandey A. Biogenic nanoparticles as safer alternatives for gastric ulcers: an update on green synthesis methods, toxicity, and their efficacy in controlling inflammation. Biol Trace Elem Res. (2024). 10.1007/s12011-024-04446-4 39570521

[3] Rajkumar C Ramsridhar S Veeraraghavan VP Francis AP Purushotham M Mageshwari U. Anticancer effect of Moringa Oleifera in oral squamous cell carcinoma: a systematic review. Discov Oncol. (2024) 15(1):688. 10.1007/s12672-024-01557-1 39570553 PMC11582275

[4] González Y Mojica-Flores R Moreno-Labrador D Pecchio M Rao KSJ Ahumedo-Monterrosa M Tetrahydrocurcumin derivatives enhanced the anti-inflammatory activity of curcumin: synthesis, biological evaluation, and structure–activity relationship analysis. Molecules. (2023) 28(23):7787. 10.3390/molecules28237787 38067518 PMC10708537

[5] Zhou M Li R Hua H Dai Y Yin Z Li L The role of tetrahydrocurcumin in disease prevention and treatment. Food Funct. (2024) 15(13):6798–824. 10.1039/D3FO05739A 38836693

[6] Sandur SK Pandey MK Sung B Ahn KS Murakami A Sethi G Curcumin, demethoxycurcumin, bisdemethoxycurcumin, tetrahydrocurcumin and turmerones differentially regulate anti-inflammatory and anti-proliferative responses through a ROS-independent mechanism. Carcinogenesis. (2007) 28(8):1765–73. 10.1093/carcin/bgm123 17522064

[7] Li J Sesso HD Kim E Manson JE Friedenberg G Clar A Cocoa extract supplementation and risk of type 2 diabetes: the cocoa supplement and multivitamin outcomes study (COSMOS) randomized clinical trial. Diabetes Care. (2023) 46(12):2278–84. 10.2337/dc23-1012 37816167 PMC10698212

[8] Luo J Zhou L Sun A Yang H Zhang P Liu K Herbal medicine for Hashimoto’s thyroiditis: a systematic review and network meta-analysis. J Ethnopharmacol. (2024) 323:117663. 10.1016/j.jep.2023.117663 38181936

[9] Huang C-C Narayanan R Warshawsky N Ravindran S. Dual ECM biomimetic scaffolds for dental pulp regenerative applications. Front Physiol. (2018) 9:495. 10.3389/fphys.2018.00495 29887803 PMC5981804

[10] Matoug-Elwerfelli M Nazzal H Raif EM Wilshaw S-P Esteves F Duggal M. Ex-vivo recellularisation and stem cell differentiation of a decellularised rat dental pulp matrix. Sci Rep. (2020) 10(1):21553. 10.1038/s41598-020-78477-x 33299073 PMC7725831

[11] Chen R Wang M Qi Q Tang Y Guo Z Wu S Sequential anti-inflammatory and osteogenic effects of a dual drug delivery scaffold loaded with parthenolide and naringin in periodontitis. J Periodontal Implant Sci. (2022) 53:20–37. 10.5051/jpis.2105700285 36468470 PMC9943701

[12] Liu J Ruan J Weir MD Ren K Schneider A Wang P Periodontal bone-ligament-cementum regeneration via scaffolds and stem cells. Cells. (2019) 8(6):537. 10.3390/cells8060537 31167434 PMC6628570

[13] Ledesma-Martínez E Mendoza-Núñez VM Santiago-Osorio E. Mesenchymal stem cells for periodontal tissue regeneration in elderly patients. J Gerontol Series A. (2019) 74(9):1351–8. 10.1093/gerona/gly227 30289440

[14] Xu X-Y Li X Wang J He X-T Sun H-H Chen F-M. Concise review: periodontal tissue regeneration using stem cells: strategies and translational considerations. Stem Cells Transl Med. (2019) 8(4):392–403. 10.1002/sctm.18-0181 30585445 PMC6431686

[15] Shi H Zong W Xu X Chen J. Improved biphasic calcium phosphate combined with periodontal ligament stem cells may serve as a promising method for periodontal regeneration. Am J Transl Res. (2018) 10(12):4030–41. 30662648 PMC6325501

[16] Du J Shan Z Ma P Wang S Fan Z. Allogeneic bone marrow mesenchymal stem cell transplantation for periodontal regeneration. J Dent Res. (2014) 93(2):183–8. 10.1177/0022034513513026 24226426

[17] Bose S Sarkar N. Natural medicinal compounds in bone tissue engineering. Trends Biotechnol. (2020) 38(4):404–17. 10.1016/j.tibtech.2019.11.005 31882304 PMC8015414

[18] Rodriguez I Conti T Bionda N. A preliminary direct comparison of the inflammatory reduction and growth factor production capabilities of three commercially available wound products: collagen sheet, manuka honey sheet, and a novel bioengineered collagen derivative+manuka honey+hydroxyapatite sheet. Int J Mol Sci. (2022) 23(18):10670. 10.3390/ijms231810670 36142583 PMC9503338

[19] Santos JAA Da Silva JW Dos Santos SM Rodrigues MDF Silva CJA Da Silva MV *In vitro* and *in vivo* wound healing and anti-inflammatory activities of babassu oil (Attalea speciosa mart. ex spreng., arecaceae). Evidence-Based Complementary Altern Med. (2020) 2020:8858291. 10.1155/2020/8858291 33029179 PMC7532363

[20] Azadbakht M Asghari M Dailami KN Davoodi A Ahmadi A. The preventive effects of Asparagus officinalis extract on sodium selenite-induced cataractogenesis in experimental animal models. Evid Based Complement Alternat Med. (2020) 2020:3708730. 10.1155/2020/3708730 33299449 PMC7704146

[21] Baptista AB Sarandy MM Gonçalves RV Novaes RD Gonçalves Da Costa C Leite JPV Antioxidant and anti-inflammatory effects of Anacardium occidentale L. and Anacardium microcarpum D. Extracts on the liver of IL-10 knockout mice. Evid Based Complement Alternat Med. (2020) 2020:3054521. 10.1155/2020/3054521 33376496 PMC7744185

[22] Shinkre R Rodrigues E Mukherji I Pandya D Naik R Banerjee A. Cissus extracts in dentistry: a comprehensive review on its untapped potential. J Pharm Bioallied Sci. (2024) 16(1):S60–2. 10.4103/jpbs.jpbs_976_23 38595361 PMC11000864

[23] Hashim NT Babiker R Rahman MM Mohamed R Priya SP Chaitanya NC Natural bioactive compounds in the management of periodontal diseases: a comprehensive review. Molecules. (2024) 29(13):3044. 10.3390/molecules29133044 38998994 PMC11242977

[24] Atanasova-Panchevska N Stojchevski R Hadzi-Petrushev N Mitrokhin V Avtanski D Mladenov M. Antibacterial and antiviral properties of tetrahydrocurcumin-based formulations: an overview of their metabolism in different microbiotic compartments. Life. (2022) 12(11):1708. 10.3390/life12111708 36362863 PMC9696410

[25] Zhang L Li C Wang S Avtanski D Hadzi-Petrushev N Mitrokhin V Tetrahydrocurcumin-related vascular protection: an overview of the findings from animal disease models. Molecules. (2022) 27(16):5100. 10.3390/molecules27165100 36014335 PMC9412611

[26] Aggarwal B Deb L Prasad S. Curcumin differs from tetrahydrocurcumin for molecular targets, signaling pathways and cellular responses. Molecules. (2014) 20(1):185–205. 10.3390/molecules20010185 25547723 PMC6272158

[27] Langmead B Salzberg SL. Fast gapped-read alignment with Bowtie 2. Nat Methods. (2012) 9(4):357–9. 10.1038/nmeth.1923 22388286 PMC3322381

[28] Dobin A Davis CA Schlesinger F Drenkow J Zaleski C Jha S STAR: ultrafast universal RNA-seq aligner. Bioinformatics. (2013) 29(1):15–21. 10.1093/bioinformatics/bts635 23104886 PMC3530905

[29] Love MI Huber W Anders S. Moderated estimation of fold change and dispersion for RNA-seq data with DESeq2. Genome Biol. (2014) 15(12):550. 10.1186/s13059-014-0550-8 25516281 PMC4302049

[30] Birjandi A Sharpe P. Exploring the therapeutic potential of curcumin for periodontal regeneration. Dent Adv Res. (2024) 9:207. 10.29011/2574-7347.100207

[31] Beurel E Grieco SF Jope RS. Glycogen synthase kinase-3 (GSK3): regulation, actions, and diseases. Pharmacol Ther. (2015) 148:114–31. 10.1016/j.pharmthera.2014.11.016 25435019 PMC4340754

[32] Fuerer C Nusse R ten Berge D. Wnt signalling in development and disease: Max Delbrück Center for Molecular Medicine Meeting on Wnt signaling in development and disease. EMBO Rep. (2008) 9(2):134–8. 10.1038/sj.embor.7401159 18188179 PMC2246409

[33] Liu F Millar SE. Wnt/beta-catenin signaling in oral tissue development and disease. J Dent Res. (2010) 89(4):318–30. 10.1177/0022034510363373 20200414 PMC3140915

[34] Maretto S Cordenonsi M Dupont S Braghetta P Broccoli V Hassan AB Mapping Wnt/beta-catenin signaling during mouse development and in colorectal tumors. Proc Natl Acad Sci U S A. (2003) 100(6):3299–304. 10.1073/pnas.0434590100 12626757 PMC152286

[35] Yuan X Pei X Zhao Y Tulu US Liu B Helms JA. A Wnt-responsive PDL population effectuates extraction socket healing. J Dent Res. (2018) 97(7):803–9. 10.1177/0022034518755719 29420105 PMC6728586

[36] Ouspenskaia T Matos I Mertz AF Fiore VF Ouspenskaia T Matos I WNT-SHH antagonism specifies and expands stem cells prior to niche formation article WNT-SHH antagonism specifies and expands stem cells prior to niche formation. Cell. (2016) 164(1–2):156–69. 10.1016/j.cell.2015.11.058 26771489 PMC4850916

[37] Han P Ivanovski S Crawford R Xiao Y. Activation of the canonical Wnt signaling pathway induces cementum regeneration. J Bone Miner Res. (2015) 30(7):1160–74. 10.1002/jbmr.2445 25556853

[38] Hunter DJ Bardet C Mouraret S Liu B Singh G Sadoine J Wnt acts as a prosurvival signal to enhance dentin regeneration. J Bone Miner Res. (2015) 30(7):1150–9. 10.1002/jbmr.2444 25556760

[39] Zhang P Liu H Yu Y Peng S Zhu S. Role of Curcuma longae rhizoma in medical applications: research challenges and opportunities. Front Pharmacol. (2024) 15:1430284. 10.3389/fphar.2024.1430284 39170702 PMC11336575

[40] Ma Y Ye S Sun K Gu Y. Effect of curcumin nanoparticles on proliferation and migration of mouse airway smooth muscle cells and airway inflammatory infiltration. Front Pharmacol. (2024) 15:1344333. 10.3389/fphar.2024.1344333 38708080 PMC11066239

[41] Jena KK Mehto S Kolapalli SP Nath P Chauhan S Chauhan S. TRIM16 Employs NRF2, ubiquitin system and aggrephagy for safe disposal of stress-induced misfolded proteins. Cell Stress. (2018) 2(12):365–7. 10.15698/cst2018.12.169 31225461 PMC6551674

[42] Bell JL Malyukova A Holien JK Koach J Parker MW Kavallaris M TRIM16 acts as an E3 ubiquitin ligase and can heterodimerize with other TRIM family members. PLoS One. (2012) 7(5):e37470. 10.1371/journal.pone.0037470 22629402 PMC3357404

[43] Liu H Povysheva N Rose ME Mi Z Banton JS Li W Role of UCHL1 in axonal injury and functional recovery after cerebral ischemia. Proc Natl Acad Sci U S A. (2019) 116(10):4643–50. 10.1073/pnas.1821282116 30760601 PMC6410860

[44] Coudert AE Del Fattore A Baulard C Olaso R Schiltz C Collet C Differentially expressed genes in autosomal dominant osteopetrosis type II osteoclasts reveal known and novel pathways for osteoclast biology. Lab Invest. (2014) 94(3):275–85. 10.1038/labinvest.2013.140 24336069

[45] Shim S Kwon Y-B Yoshikawa Y Kwon J. Ubiquitin C-terminal hydrolase L1 deficiency decreases bone mineralization. J Vet Med Sci. (2008) 70(6):649–51. 10.1292/jvms.70.649 18628613

[46] Chazenbalk G Chen Y-H Heneidi S Lee J-M Pall M Chen Y-DI Abnormal expression of genes involved in inflammation, lipid metabolism, and Wnt signaling in the adipose tissue of polycystic ovary syndrome. J Clin Endocrinol Metab. (2012) 97(5):E765–70. 10.1210/jc.2011-2377 22344199 PMC3339894

[47] Bouron A Aubry L Loreth D Fauvarque M-O Meyer-Schwesinger C. Role of the deubiquitinating enzyme UCH-L1 in mitochondrial function. Front Cell Neurosci. (2023) 17:1149954. 10.3389/fncel.2023.1149954 37032833 PMC10076731

[48] Zheng F Wang F Wu T Tang H Li H Cui X Ubiquitin C-terminal hydrolase L1 activation in periodontal ligament cells mediates orthodontic tooth movement via the MAPK signaling pathway. Connect Tissue Res. (2024) 65(5):421–32. 10.1080/03008207.2024.2395998 39221694

[49] Damgaard RB. The ubiquitin system: from cell signalling to disease biology and new therapeutic opportunities. Cell Death Differ. (2021) 28(2):423–6. 10.1038/s41418-020-00703-w 33446876 PMC7862391

[50] Tsuchida S Nakayama T. Ubiquitination and deubiquitination in oral disease. Int J Mol Sci. (2021) 22(11):5488. 10.3390/ijms22115488 34070986 PMC8197098

[51] Jena KK Kolapalli SP Mehto S Nath P Das B Sahoo PK TRIM16 controls assembly and degradation of protein aggregates by modulating the P62-NRF2 axis and autophagy. EMBO J. (2018) 37(18):e98358. 10.15252/embj.201798358 30143514 PMC6138442

[52] Zhang W Shen J Zhang S Liu X Pan S Li Y Silencing integrin *Α*6 enhances the pluripotency–differentiation transition in human dental pulp stem cells. Oral Dis. (2022) 28(3):711–22. 10.1111/odi.13771 33404136

[53] Zhu Q Safavi KE Spångberg LSW. Integrin expression in human dental pulp cells and their role in cell attachment on extracellular matrix proteins. J Endod. (1998) 24(10):641–4. 10.1016/S0099-2399(98)80145-3 10023243

[54] Wang H Ning T Song C Luo X Xu S Zhang X Priming integrin *Α*5 promotes human dental pulp stem cells odontogenic differentiation due to extracellular matrix deposition and amplified extracellular matrix-receptor activity. J Cell Physiol. (2019) 234(8):12897–909. 10.1002/jcp.27954 30556904 PMC13483343

[55] Chen L Wang X Tian S Zhou L Wang L Liu X Integrin-linked kinase control dental pulp stem cell senescence via the MTOR signaling pathway. Stem Cells. (2024) 42(10):861–73. 10.1093/stmcls/sxae047 39169713 PMC11464141

[56] Zhao Y Wang L Jin Y Shi S. Fas ligand regulates the immunomodulatory properties of dental pulp stem cells. J Dent Res. (2012) 91(10):948–54. 10.1177/0022034512458690 22904205 PMC3446835

[57] Pisciotta A Bertani G Bertoni L Di Tinco R De Biasi S Vallarola A Modulation of cell death and promotion of chondrogenic differentiation by Fas/FasL in human dental pulp stem cells (HDPSCs). Front Cell Dev Biol. (2020) 8:279. 10.3389/fcell.2020.00279 32500073 PMC7242757

[58] Kim J-H Irfan M Hossain MA George A Chung S. BDNF/Trkb is a crucial regulator in the inflammation-mediated odontoblastic differentiation of dental pulp stem cells. Cells. (2023) 12(14):1851. 10.3390/cells12141851 37508514 PMC10378460

[59] Hucklenbroich J Klein R Neumaier B Graf R Fink GR Schroeter M Aromatic-turmerone induces neural stem cell proliferation *in vitro* and *in vivo*. Stem Cell Res Ther. (2014) 5(4):100. 10.1186/scrt500 25928248 PMC4180255

[60] Mohammadi R Mahmoodi H. Improvement of peripheral nerve regeneration following nerve repair by silicone tube filled with curcumin: a preliminary study in the rat model. Int J Surg. (2013) 11(9):819–25. 10.1016/j.ijsu.2013.08.011 23994006

[61] Wang Y Lin H Huang W Liu Z Chen Z Zhao X Curcumin attenuates periodontal injury via inhibiting ferroptosis of ligature-induced periodontitis in mice. Int J Mol Sci. (2023) 24(12):9835. 10.3390/ijms24129835 37372983 PMC10298010

[62] Verma SS Avadhesh Srivastava A Shekher A Dhasmana A Narula AS Evaluation of efficacy of curcumin and caffeic acid phenethyl ester in breast cancer by preclinical studies. Curr Med Chem. (2024) 31. 10.2174/0109298673284600231230093955 38523543

[63] Ye H Li Z Yang J Long Y Zhong Y Wu Y A network pharmacology-based study to investigate the mechanism of curcumin-regulated regenerative repair of quadriceps femoris muscle in KOA rats. Eur J Pharmacol. (2024) 982:176910. 10.1016/j.ejphar.2024.176910 39154821

[64] Gouda MM Balaya RDA Modi PK Kadri S Chanderasekaran J Balnadupete A Impact of curcumin on the IL-17A-mediated P53-fibrinolytic system: mouse proteomics and integrated human fibrosis ScRNAseq insights. Inflammation. (2024). 10.1007/s10753-024-02167-3 39424752

[65] Dasi D Nallabelli N Devalaraju R S KN Ghosh S Karnati R Curcumin attenuates replicative senescence in human dental follicle cells and restores their osteogenic differentiation. J Oral Biosci. (2023) 65(4):371–8. 10.1016/j.job.2023.10.001 37806337

[66] Yang Z He C He J Chu J Liu H Deng X. Curcumin-mediated bone marrow mesenchymal stem cell sheets create a favorable immune microenvironment for adult full-thickness cutaneous wound healing. Stem Cell Res Ther. (2018) 9(1):1–18. 10.1186/s13287-018-0768-6 29386050 PMC5793416

[67] Lan C Qian Y Wang Y Chen Y Lin C Zhang Y The protective role of curcumin in human dental pulp stem cells stimulated by lipopolysaccharide via inhibiting NF-ΚB P65 phosphorylation to suppress NLRP3 inflammasome activation. Clin Oral Investig. (2023) 27(6):2875–85. 10.1007/s00784-023-04885-8 36735089

[68] Ameer SF Mohamed MY Elzubair QA Sharif EAM Ibrahim WN. Curcumin as a novel therapeutic candidate for cancer: can this natural compound revolutionize cancer treatment? Front Oncol. (2024) 14:1438040. 10.3389/fonc.2024.1438040 39507759 PMC11537944

[69] Xiao Y Dai Y Li L Geng F Xu Y Wang J Tetrahydrocurcumin ameliorates Alzheimer’s pathological phenotypes by inhibition of microglial cell cycle arrest and apoptosis via Ras/ERK signaling. Biomed Pharmacother. (2021) 139:111651. 10.1016/j.biopha.2021.111651 34243602

[70] Pan Y Zhang Y Yuan J Ma X Zhao Y Li Y Tetrahydrocurcumin mitigates acute hypobaric hypoxia-induced cerebral oedema and inflammation through the NF-*κ*B/VEGF/MMP-9 pathway. Phytother Res. (2020) 34(11):2963–77. 10.1002/ptr.6724 32573860

[71] Wu Y Chen Y Li Q Ye X Guo X Sun L Tetrahydrocurcumin alleviates allergic airway inflammation in asthmatic mice by modulating the gut microbiota. Food Funct. (2021) 12(15):6830–40. 10.1039/D1FO00194A 34116562

[72] Li L Liu X Li S Wang Q Wang H Xu M Tetrahydrocurcumin protects against sepsis-induced acute kidney injury via the SIRT1 pathway. Ren Fail. (2021) 43(1):1028–40. 10.1080/0886022X.2021.1942915 34187277 PMC8253188

[73] Yoysungnoen B Srisawat U Piyabhan P Duansak N Sookprasert N Mathuradavong N Short term effect of tetrahydrocurcumin on adipose angiogenesis in very high-fat diet-induced obesity mouse model. Front Nutr. (2023) 10:1221935. 10.3389/fnut.2023.1221935 37876615 PMC10591188

[74] Zhang Y Liu Y Zou J Yan L Du W Zhang Y Tetrahydrocurcumin induces mesenchymal-epithelial transition and suppresses angiogenesis by targeting HIF-1*α* and *Autophagy* in human osteosarcoma. Oncotarget. (2017) 8(53):91134–49. 10.18632/oncotarget.19845 29207631 PMC5710911

[75] Wang Z Dabrosin C Yin X Fuster MM Arreola A Rathmell WK Broad targeting of angiogenesis for cancer prevention and therapy. Semin Cancer Biol. (2015) 35:S224–43. 10.1016/j.semcancer.2015.01.001 25600295 PMC4737670

