# Supplementary material for: Therapeutic potential of curcumin in regenerative dentistry
Source: Front Dent Med. 2025 Mar 24;6:1537478. doi: 10.3389/fdmed.2025.1537478 (PMC11973315; doi:10.3389/fdmed.2025.1537478)
Supplement: Supplementary file 1 [file Image1.pdf]

Supplementary Material

Supplementary Figure

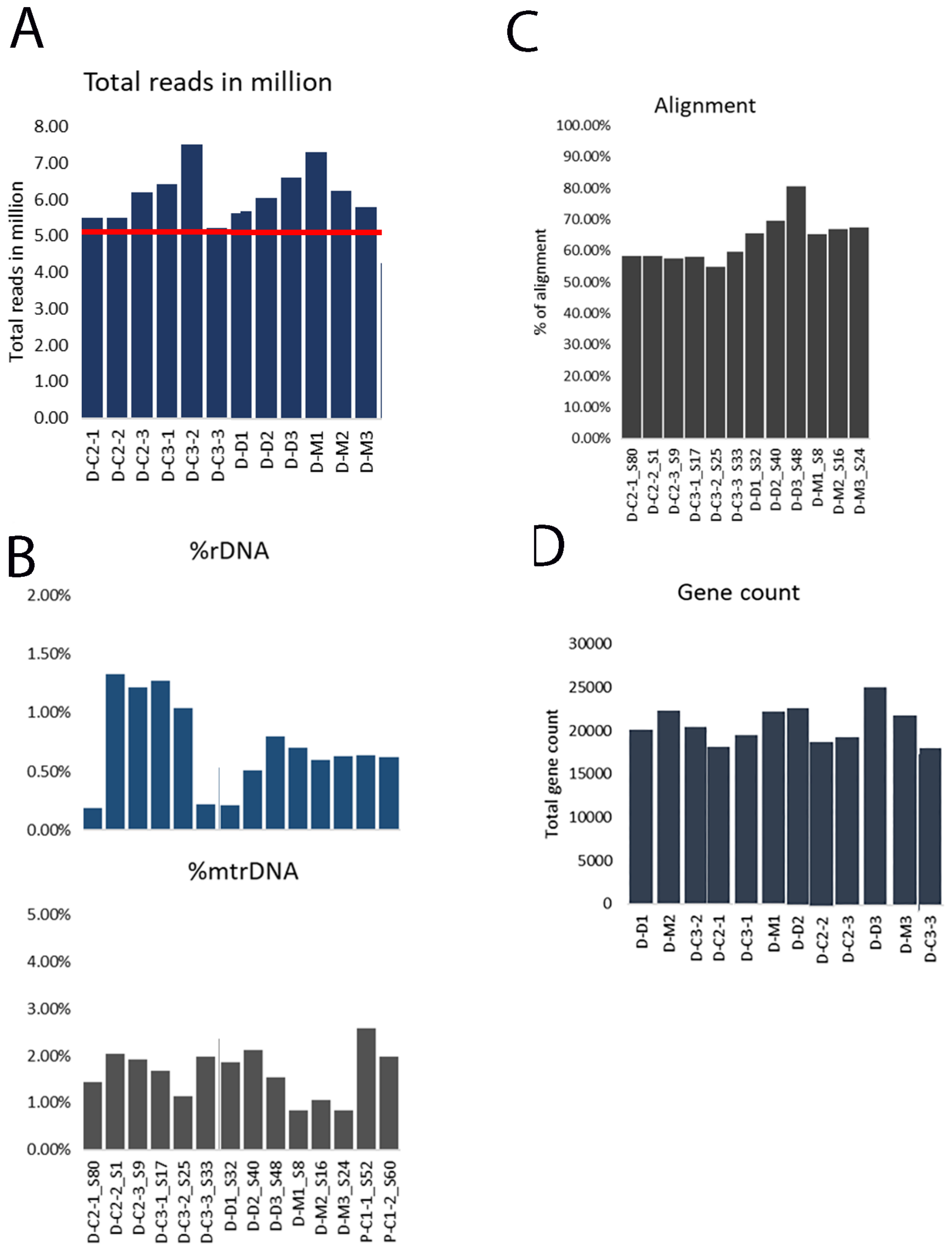

Supplementary Figure 1. Bulk Sequencing of dental pulp cells after treatment with curcumin and Tetrahydrocurcumin. Total reads (A), Ribosomal and mitochondrial RNA contamination with <3% ribosomal and mitochondrial RNA suggesting good quality samples (B). %alignment (c)Number of genes that were detected per samples(D). Triplicates of each sample group. D: DMSO, M: Media C2; curcumin, C3: tetrahydrocurcumin,
